# Assessment of the viability of integrating virtual reality programs in practical tests for the Korean Radiological Technologists Licensing Examination: a survey study

**DOI:** 10.3352/jeehp.2023.20.33

**Published:** 2023-11-28

**Authors:** Hye Min Park, Eun Seong Kim, Deok Mun Kwon, Pyong Kon Cho, Seoung Hwan Kim, Ki Baek Lee, Seong Hu Kim, Moon Il Bong, Won Seok Yang, Jin Eui Kim, Gi Bong Kang, Yong Su Yoon, Jung Su Kim

**Affiliations:** 1 Department of Radiology, Masan University, Changwon, Korea; 2 Department of Radiology, Seoul National University Hospital, Seoul, Korea; 3 Department of Radiological Technology, Daegu Health College, Daegu, Korea; 4 Department of Radiological Science, Daegu Catholic University, Daegu, Korea; 5 Department of Radiological Science, College of Health Medical Science, Cheongju University, Cheongju, Korea; 6 Department of Radiologic Technology, Chungbuk Health & Science University, Cheongju, Korea; 7 Department of Radiological Technology, Gwangju Health University, Gwangju, Korea; 8 Department of Radiology, Dong-A University Hospital, Busan, Korea; 9 Department of Nuclear Medicine, Seoul National University Hospital, Seoul, Korea; 10 Department of Radiology, Yonsei University College of Medicine, Seoul, Korea; 11 Department of Radiological Science, Dongseo University, Busan, Korea; Hallym University, Korea

**Keywords:** Virtual reality, Augmented reality, Mixed reality, Radiologic technologist, Qualify examination

## Abstract

**Purpose:**

The objective of this study was to assess the feasibility of incorporating virtual reality/augmented reality (VR/AR) programs into practical tests administered as part of the Korean Radiological Technologists Licensing Examination (KRTLE). This evaluation is grounded in a comprehensive survey that targeted enrolled students in departments of radiology across the nation.

**Methods:**

In total, 682 students from radiology departments across the nation were participants in the survey. An online survey platform was used, and the questionnaire was structured into 5 distinct sections and 27 questions. A frequency analysis for each section of the survey was conducted using IBM SPSS ver. 27.0.

**Results:**

Direct or indirect exposure to VR/AR content was reported by 67.7% of all respondents. Furthermore, 55.4% of the respondents expressed that VR/AR could be integrated into their classes, which signified a widespread acknowledgment of VR among the students. With regards to the integration of a VR/AR or mixed reality program into the practical tests for purposes of the KRTLE, a substantial amount of the respondents (57.3%) exhibited a positive inclination and recommended its introduction.

**Conclusion:**

The application of VR/AR programs within practical tests of the KRTLE will be used as an alternative for evaluating clinical examination procedures and validating job skills.

## Graphical abstract

[Fig f7-jeehp-20-33]

## Introduction

### Background/rationale

To qualify as a radiological technologist (RT) in Korea, individuals are required to pass an examination that evaluates their knowledge and skills [1]. This examination is referred to as the Korean Radiological Technologists Licensing Examination (KRTLE). The KRTLE assesses fundamental medical theory and the practical application of radiation. It is structured as written and practical tests [1,2]. In the past, individuals who passed the theory test were granted permission to proceed to the practical test. However, due to the increasing number of test takers, a new approach was adopted, where the practical tests were administered through visual representations and conducted concurrently with the theory tests.

Recently, virtual reality (VR), augmented reality (AR), and mixed reality (MR) have emerged as promising methods for education and training within the medical domain. These are particularly gaining prominence in supporting medical treatment and practical training [3,4]. VR creates a computer-generated environment that immerses users within a specific scenario, involving a simulation with actual situations [5]. AR overlays 3-dimensional virtual images onto real-world visuals, effectively merging virtual elements with real-time additional information, thus providing users with a unified interface within their physical environment [6]. Lastly, MR combines the attributes of both VR and AR, facilitating real-time interactions between real and virtual objects [7]. These technologies rely on devices such as head-mounted displays to seamlessly blend elements of the real and virtual worlds [5,6].

A remarkable surge across diverse domains, including surgery and training support, has taken place concerning medical VR [3,4,8-10]. A domestic hospital has introduced VR-simulator development designed to create a virtual environment conducive to sinus surgery training. This approach permits the simulation of endoscopic surgery based on a patient’s high-resolution computed tomography (CT) scan, thereby enabling the practice of surgical procedures akin to real-life scenarios [8]. Moreover, the usability of a VR simulator in the dentistry licensing examination has been assessed through research conducted by the Korea Health Personnel Licensing Examination Institute [3].

### Objectives

There was originally an intention to adopt an objective structured clinical examination for the KRTLE; however, it faced limitations and, thus, the decision was made to provide photographic examples [11]. The present format, however, inevitably possesses limitations in assessing practical skills. There has recently been a surge in efforts to leverage VR programs for educational and assessment purposes in the healthcare and medical fields. The exploration of potentially integrating VR programs into the practical tests bears the promise of nurturing RTs equipped with both practical prowess and problem-solving capabilities. Thus, the objective of this study was to gather the opinions of students in departments of radiology through a survey and assess the feasibility of incorporating VR programs into the practical test of the KRTLE.

## Methods

### Ethics statement

This study was granted an exemption from research ethics review due to its nature as a survey study, in accordance with the regulations of the Institutional Review Board of the Daegu Health College (DHCIRB-2000-12-0015). Prior consent was obtained from students to utilize the survey results for research purposes.

### Study design

This study was designed as a survey study involving simple observations.

### Setting

To facilitate the survey process, an online questionnaire was generated. The online link was shared with professors at universities. The responses were collected from May 1 to 31, 2023.

### Participants

The participants were 9,298 students enrolled in 44 radiology departments in universities across the nation. The students participated in the survey voluntarily.

### Variables

The demographic characteristics of the students, such as gender and current academic year, could be potential variables. However, in this study, frequency analysis was conducted for each question, rather than conducting an analysis based on variables.

### Data sources/measurement

The questionnaire was structured into 5 distinct sections (Supplement 1). In the first section, the respondents were provided with the survey’s objectives, and the concepts of VR, AR, MR, and computer-based test (CBT). CBT refers to an assessment methodology that leverages a wired network-enabled approach, enabling test administration, scoring, and grade management through computer-based systems [12].

The second section was designed to gather information on demographic characteristics such as age, gender, educational system, current academic year, and the geographical location of universities.

The third section collected information about experiences regarding the utilization of VR programs. This section comprised 5 questions. Moreover, the survey indicated whether the respondents had encountered courses incorporating VR content. Opinions on the best type of VR programs for integration into lectures were also requested. Furthermore, the survey inquired about the anticipated positive outcomes.

The fourth section encompassed 5 questions designed to gather insights regarding the integration of VR programs into KRTLE. These questions dealt with opinions on feasibility, the most suitable types of VR for introduction, the preferred number of questions and the test format if it is to be introduced, as well as the specific subjects that respondents deemed appropriate for integration.

The fifth section was designed to collect opinions on the optimal incorporation of VR programs, within specific aspects of each subject area within the practical tests. The assessment criteria for the subjects of practical tests encompassed examination procedures and equipment used for human organs, quality control and quality assurance (QC/QA), application of contrast medium, and protocols for radiation safety management [2,11].

### Bias

Due to the nature of survey research, there might have been some selection bias. However, it is expected that the bias is undetectable since the questions were designed for simple observations.

### Study size

The total number of students considered in the study was 9,298. With a 95% confidence level and a 5% margin of error, the appropriate sample size would have been 369. However, this survey managed to exceed this threshold, with 682 voluntary respondents.

### Statistical methods

Statistical analysis was performed using IBM SPSS ver. 27.0 (IBM Corp.). The analysis focused on evaluating the frequency of responses received for each question item. Any missing values and outliers were excluded from consideration to ensure the accuracy and integrity of the results.

## Results

### Participants

A total of 682 students participated. Table 1 shows the distribution of the collected demographic characteristics. Regarding the geographical distribution, Daegu emerged as the most prevalent location, accounting for 55.7% of the respondents.

### Experience with virtual reality programs and opinions on application to subjects

**Fig. 1.**
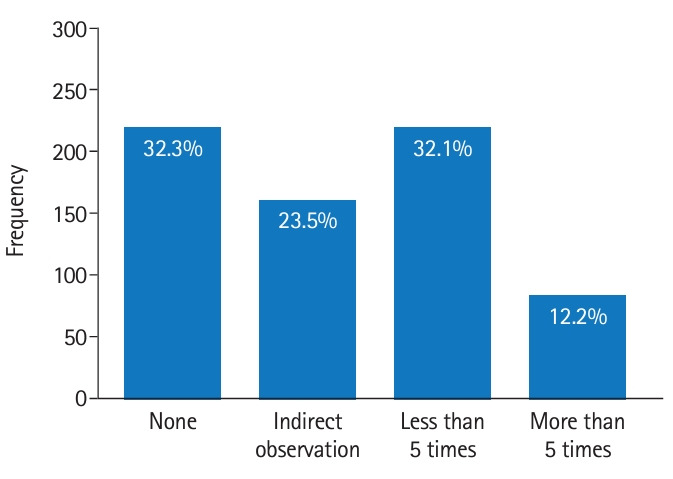
Respondents’ experiences of virtual reality content.

Fig. 1 illustrates the distribution of responses regarding VR/AR content usage experiences. In total, 8.1% of the respondents affirmed having experiences with VR content for practice subjects, while the remaining 91.9% had no such experience.

**Fig. 2.**
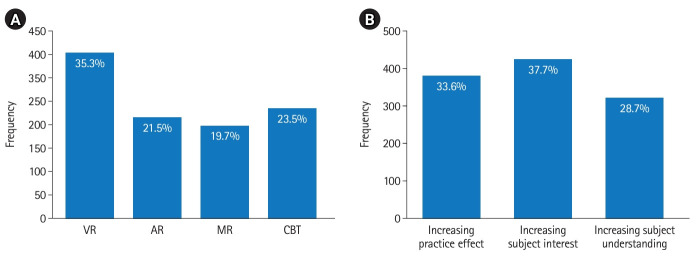
Appropriate types for applying virtual reality to classes (A) and expected effects of applying virtual reality to classes (B). VR, virtual reality; AR, augmented reality; MR, mixed reality; CBT, computer-based test.

In response to the item inquiring about the viability of integrating VR programs into classes, 55.4% expressed that it was indeed feasible, while 9.1% believed it to be unattainable. A further 35.5% of the students had not considered this aspect. A total of 1,125 responses concerning the preferred application type for classes were collected through multiple selections. Furthermore, 1,124 responses were compiled on the anticipated effects of introducing VR programs to classes. Fig. 2 indicates the distribution of these responses.

### Opinions on the introduction of virtual reality programs in the practical test

**Fig. 3.**
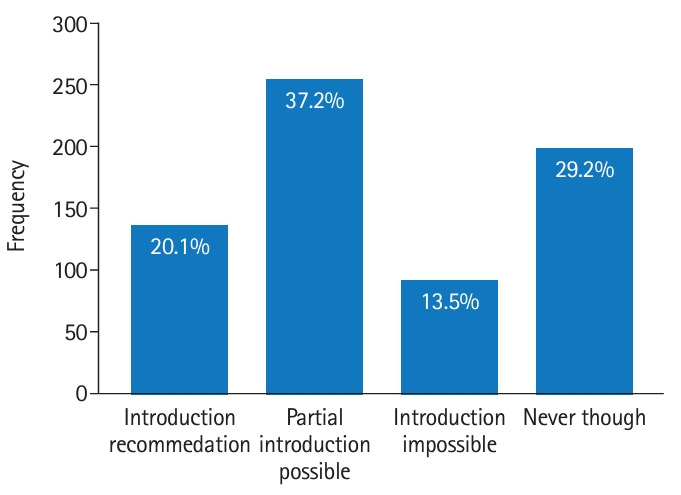
Opinion on the adoption of a virtual reality program in the practical test for the Korean Radiological Technologists Licensing Examination.

Fig. 3 illustrates the responses concerning the incorporation of VR programs into the KRTLE. A total of 1,254 responses were collected for the appropriate fields, with 24.7% of the respondents in favor of using VR for image anatomy, 23.9% in favor of using it for radiation equipment operation settings, 23.2% in favor of using it for clinical examination posture practice, 15.6% in favor of using it for radiation equipment structure, 7.9% in favor of using it for all parts of the test, and 4.7% in favor of using it for QC/QA.

**Fig. 4.**
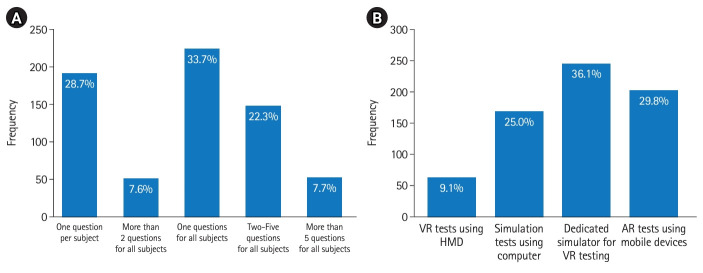
Optimal number of questions (A) and test formats (B) for introducing virtual reality (VR) to the practical test for the Korean Radiological Technologists Licensing Examination. HMD, head-mounted display; AR, augmented reality.

Regarding the question pertaining the suitable number of questions if a VR program is implemented for the practical test, the distribution of responses is displayed in Fig. 4A, while Fig. 4B illustrates the distribution of the responses concerning the suitable method.

Regarding the 3 subjects for which VR programs would be most appropriate in the practical test, a total of 1,408 responses were analyzed. Upon combining the responses from the same field, it was found that 255 students indicated subjects related to anatomy as being the most suitable.

### Opinions on the introduction of virtual reality programs in each subject area of the practical test

**Fig. 5.**
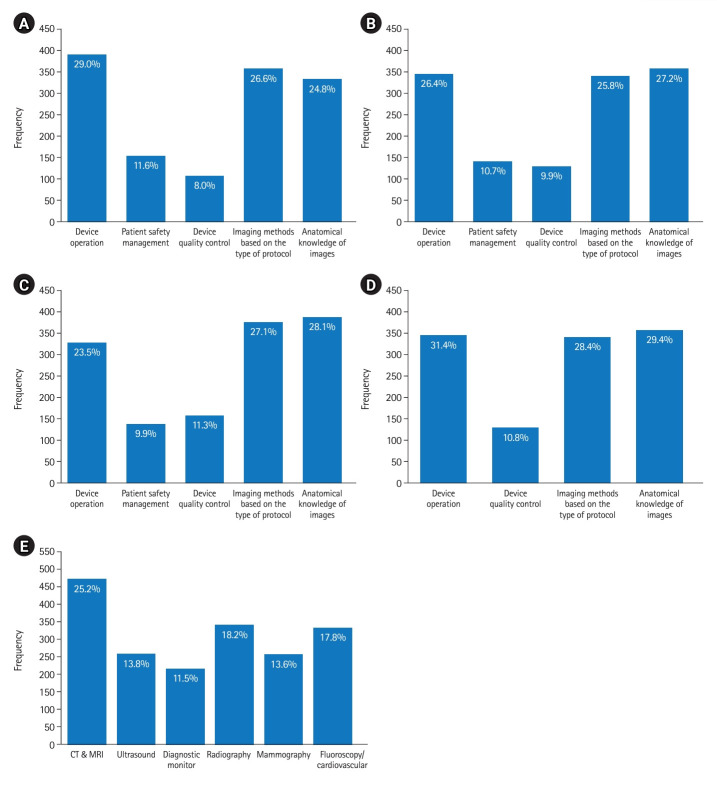
Appropriate fields for introducing virtual reality programs in radiology imaging (A), fluoroscopy (B), cardiovascular and interventional procedures (C), ultrasound technology (D), and image quality control (E) for the Korean Radiological Technologists Licensing Examination. CT, computed tomography; MRI, magnetic resonance imaging.

For each of the 9 subjects within the practical test, the respondents indicated the aspects that would be suitable for the introduction of a VR program. Multiple selections were permitted across all subjects. A total of 1,342 responses for radiological imaging and 1,308 for fluoroscopy were collected. Additionally, 1,385 responses were gathered for cardiovascular and interventional procedures, 1,283 for ultrasound technology, and 1,867 for image quality control. Fig. 5 shows the response distributions for these 5 subjects.

**Fig. 6.**
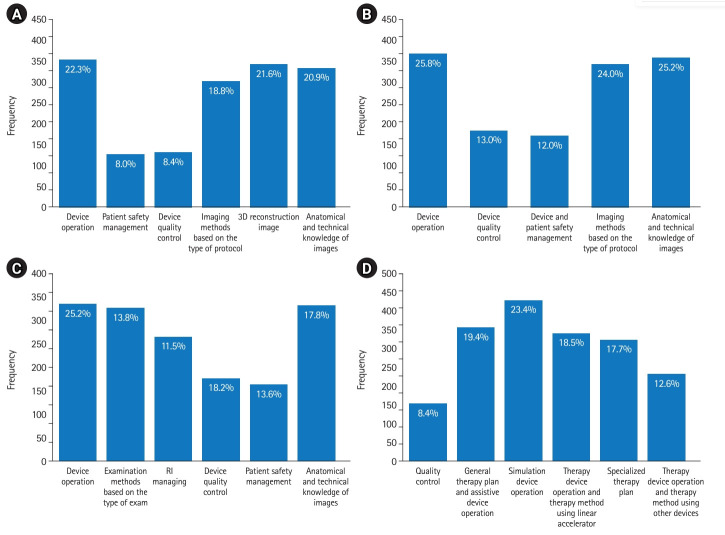
Appropriate fields for introducing virtual reality programs in computed tomography (A), magnetic resonance imaging (B), nuclear medicine technology (C), and radiation therapy (D) for the Korean Radiological Technologists Licensing Examination. 3D, 3-dimensional; RI, radioisotopes.

In total, 1,587 responses for CT, 1,419 for magnetic resonance imaging (MRI), 1,595 for nuclear medicine technology, and 1,828 for radiation therapy were collected. The response distributions for these 4 subjects are shown in Fig. 6. Students’ response data are available at Dataset 1.

## Discussion

### Key results

This study was conducted to assess the feasibility of addressing the constraints experienced in the existing practical test format of the KRTLE by substituting VR. To achieve this objective, a survey was conducted, targeting students in radiology departments.

Upon a comprehensive analysis, the incorporation of VR programs into the practical tests was shown to have validity, based on students’ questionnaire responses. Specifically, for image anatomy, the introduction of single questions drawn from a pool of practical test questions, through a simulator-based VR test, was deemed appropriate.

### Interpretation

Based on the analysis of the responses derived from the survey, more than half (55.4%) of the respondents expressed that the VR program could be integrated into their classes. Concerning the content suitable for class implementation, both VR and CBT were favored, as they are more technologically feasible than AR or MR. Numerous opinions were shared suggesting that the interest and practical benefits increase when VR programs are introduced into classes. Accordingly, it was anticipated that applying VR programs to practical subjects would yield favorable outcomes.

Pertaining to the integration of a VR program into the KRTLE, a substantial 57.3% of the respondents exhibited a positive inclination toward its introduction. The operation of radiation equipment and the clinical examination posture practice was deemed to be highly suitable for the introduction of VR programs, receiving high selection rates. In terms of the appropriate number of questions, a single item exhibited the highest responses among all 50 questions within the practical test. In relation to the type of test to be introduced, a dedicated simulator-based VR test exhibited the highest preference. Additionally, anatomy-related subjects were most frequently indicated as suitable.

Furthermore, when one question was analyzed for each subject based on the survey results, radiographic imaging, ultrasound technology, CT, MRI, and nuclear medicine technology were regarded as suitable topics in terms of device operation. The anatomical knowledge of imaging is appropriate for fluoroscopy, as well as cardiovascular and interventional procedures. Additionally, it was deemed appropriate to introduce simulator operation for radiation therapy and the QC for CT and MRI for the subject of image quality control.

Among the collected survey responses, positive feedback was given regarding “other opinions,” which indicates that the use of a VR program is appropriate for clinical training. Nonetheless, certain challenges were also suggested, such as the potential difficulty in addressing emergency scenarios during examinations due to technology instability. Furthermore, several negative responses were expressed, including concerns about fairness issues among different educational institutions.

### Comparison with previous studies

Tarutani et al. [9] explored the potential of utilizing AR for patient positioning during radiation therapy. In a study by Gunn et al. [10], the assessment of VR simulations for wrist X-ray examinations did not yield very high proficiency improvement scores. However, the study highlighted the significance of future possibilities if a tool capable of emulating real-world conditions is developed [10].

Furthermore, a previous study implemented VR modules within educational programs to assist physical therapists in clinical decision-making programs. The study’s findings demonstrated that all participants found that the VR modules were beneficial for practice. However, it should be noted that some participants indicated that while the VR-based practice was helpful, it should not replace face-to-face practice [13].

In Korea, a single instance of implementing a VR program for actual test evaluation was observed in the context of the 2022 tower crane qualification examination. Through the utilization of a VR-based tower crane operation simulator, a practical assessment was conducted as part of the National Technical Qualification Course [14].

Given the positive impact observed from the integration of VR programs into education, it is worth considering their introduction into the KRTLE, as indicated by the survey findings in this study. However, it is noted that while the integration of a VR program into an actual test has been applied in the context of the tower crane qualification test, this approach is still uncommon. Furthermore, the various perspectives shared in this study emphasize that a substantial preparatory phase is necessary to develop solutions for potential challenges before implementing VR programs.

CBT was introduced in 2022, for the first time in the context of the medical license examination, making it the inaugural instance among the 26 healthcare professional licensing examinations in Korea [15]. According to a report by Huh [15], CBT had already become an established assessment platform across numerous medical schools, thereby removing the need for an evaluation of test-taker adaptability. However, the introduction of CBT also posed a fresh challenge, which was centered around maintaining the quality of the test items and instilling them with a more clinically oriented focus, in comparison to the paper-and-pencil method [15]. Similarly, for the implementation of a VR program into the KRTLE, several key prerequisites must be addressed. First, the establishment of an educational environment capable of providing sufficient preparation for an examination at the university level is required. Second, the creation of a technically stable testing environment is essential. Lastly, there should be a determined emphasis on the establishment of a standardized evaluation system that guarantees fairness.

### Limitations

The survey targeted 9,298 students enrolled in radiology departments across the nation, but only 682 participants took part, which remains a limitation of the study. This outcome stems from encouraging voluntary participation. However, considering the confidence level (95%), it is noteworthy that the study obtained approximately 1.8 times more responses than the recommended sample size (369).

### Suggestions

Provided that adequate preparation time and a stable environment are ensured, VR programs were deemed to be an acceptable alternative to traditional practical testing. Thus, it is imperative to conduct further research involving various considerations, paving the way for potential incorporation into future trials.

### Conclusion

The current format of practical tests relies on photo-oriented assessments. However, this method struggles to effectively estimate the proficiency of clinical skills. Considering some limitations, the application of VR programs within practical tests serves as an ideal alternative for evaluating clinical examination procedures and validating job skills. Therefore, if a stable testing environment is ensured, with provisions for the availability of sufficient test opportunities, then a VR program will be considered as an asset for simulating diverse equipment scenarios and assessing students’ operational competencies.

## Figures and Tables

**Figure f7-jeehp-20-33:**
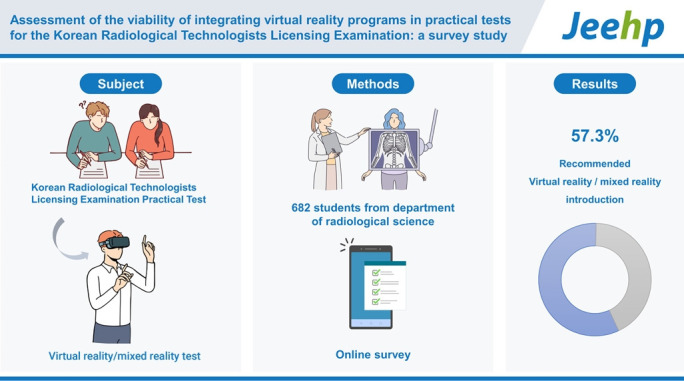


**Table 1.** Demographic characteristics of survey respondents

Characteristic	Value
Survey period	2023. 5. 1.–2023. 5. 31.
Number of participating respondents (persons)	682
Age distribution of respondents (yr)	18–53
Gender of respondents (persons)	
Male	404
Female	278
University academic system and distribution of years (persons)	
3-Year university	
1st year	151
2nd year	169
3rd year	243
4th year (advanced major)	11
4-Year university	
1st year	28
2nd year	21
3rd year	30
4th year	29

Foot notes

## Footnotes

**Data availability**
Data files are available from Harvard Dataverse: https://doi.org/10.7910/DVN/5PUZAX Dataset 1. The data file contains all university student surveys.

## Supplementary materials

Supplementary files are available from Harvard Dataverse: https://doi.org/10.7910/DVN/5PUZAX

Supplement 1. Survey questionnaires to measure the radiologic technologist students’ perceptions of the feasibility of incorporating VR programs into the practical tests of the national examination.

Supplement 2. Audio recording of the abstract.
