# Supplementary figures and images for: Assessment of the viability of integrating virtual reality programs in practical tests for the Korean Radiological Technologists Licensing Examination: a survey study
Source: J Educ Eval Health Prof. 2023 Nov 28;20:33. doi: 10.3352/jeehp.2023.20.33 (PMC10762238; doi:10.3352/jeehp.2023.20.33)

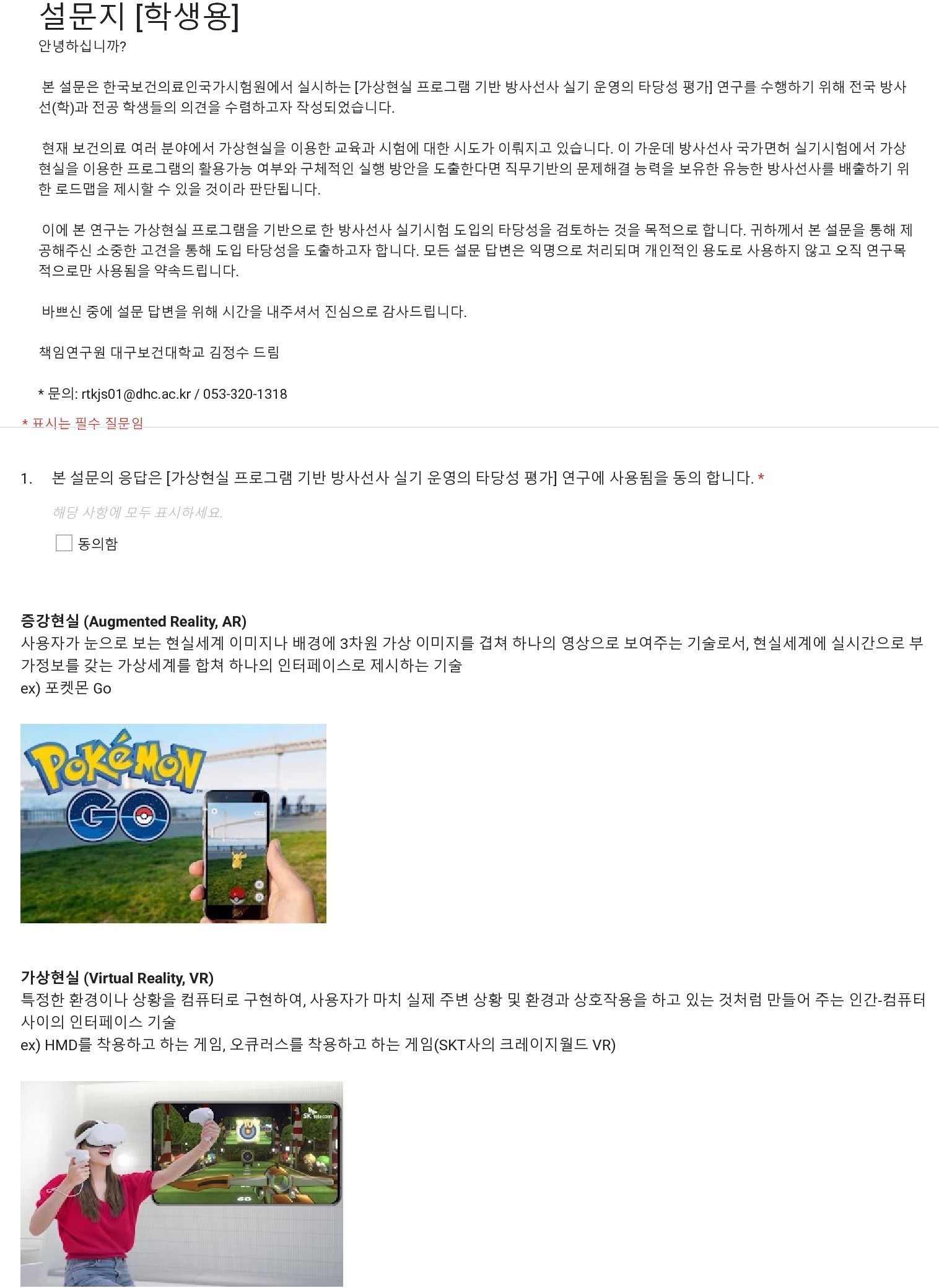


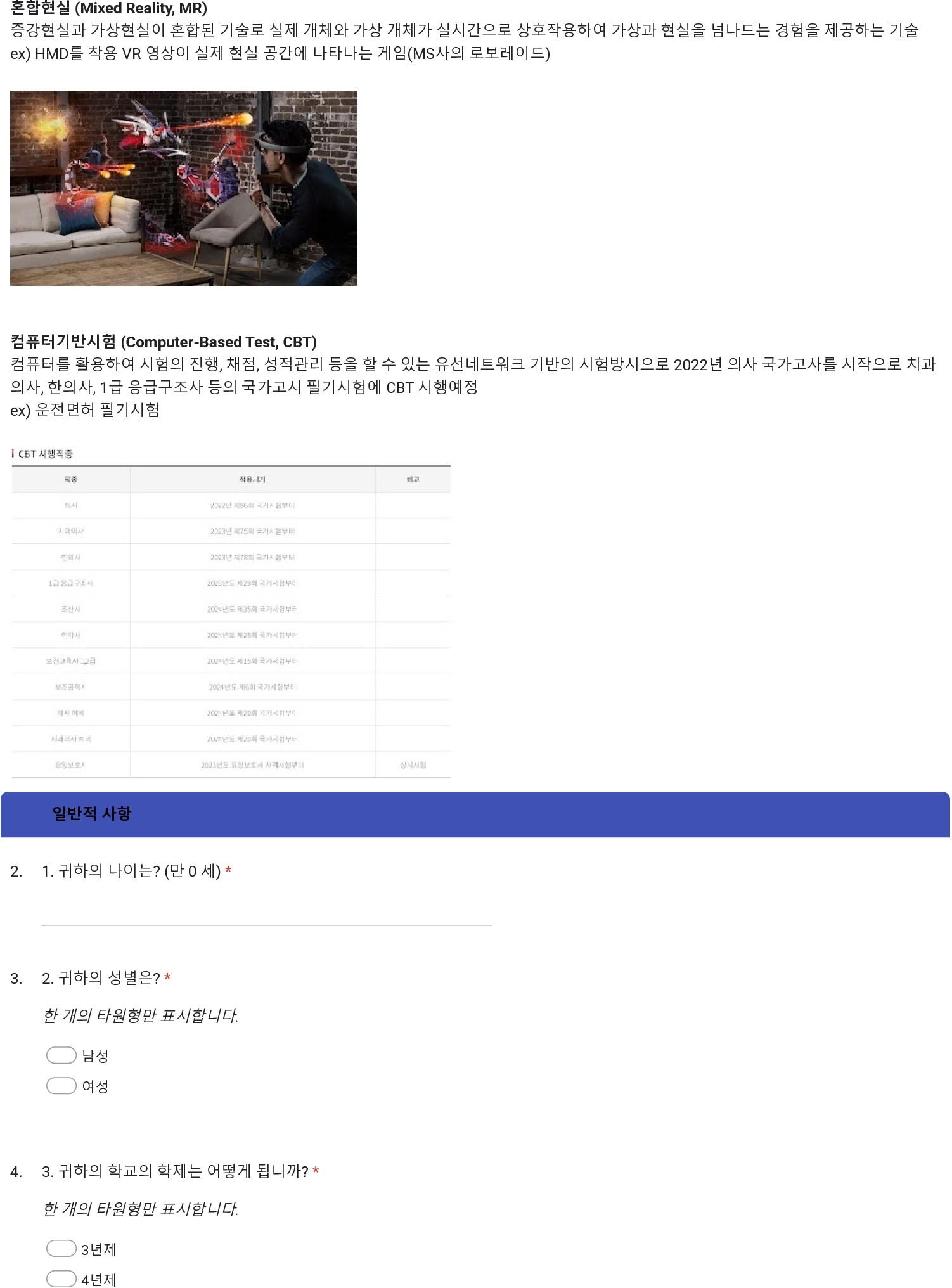


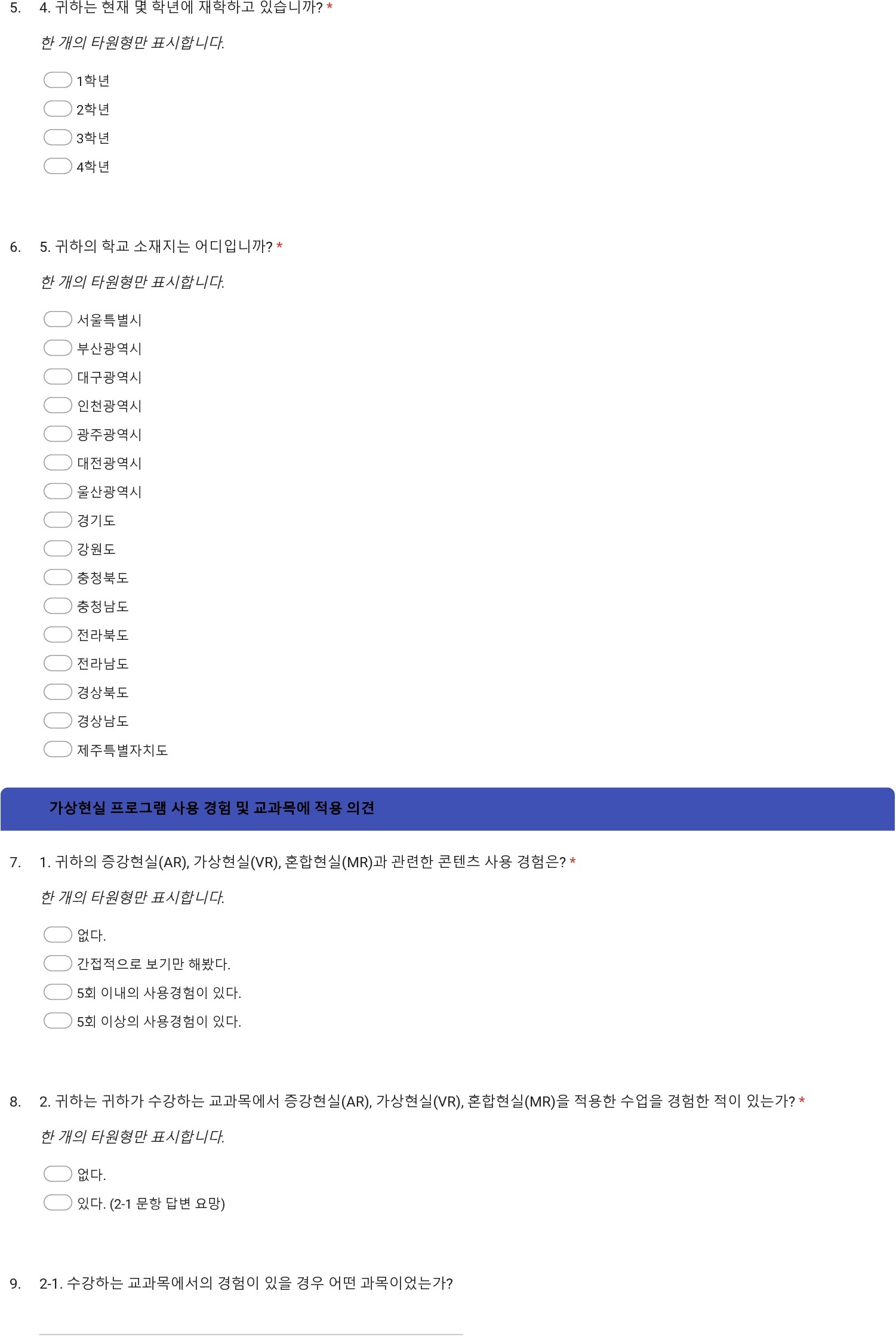


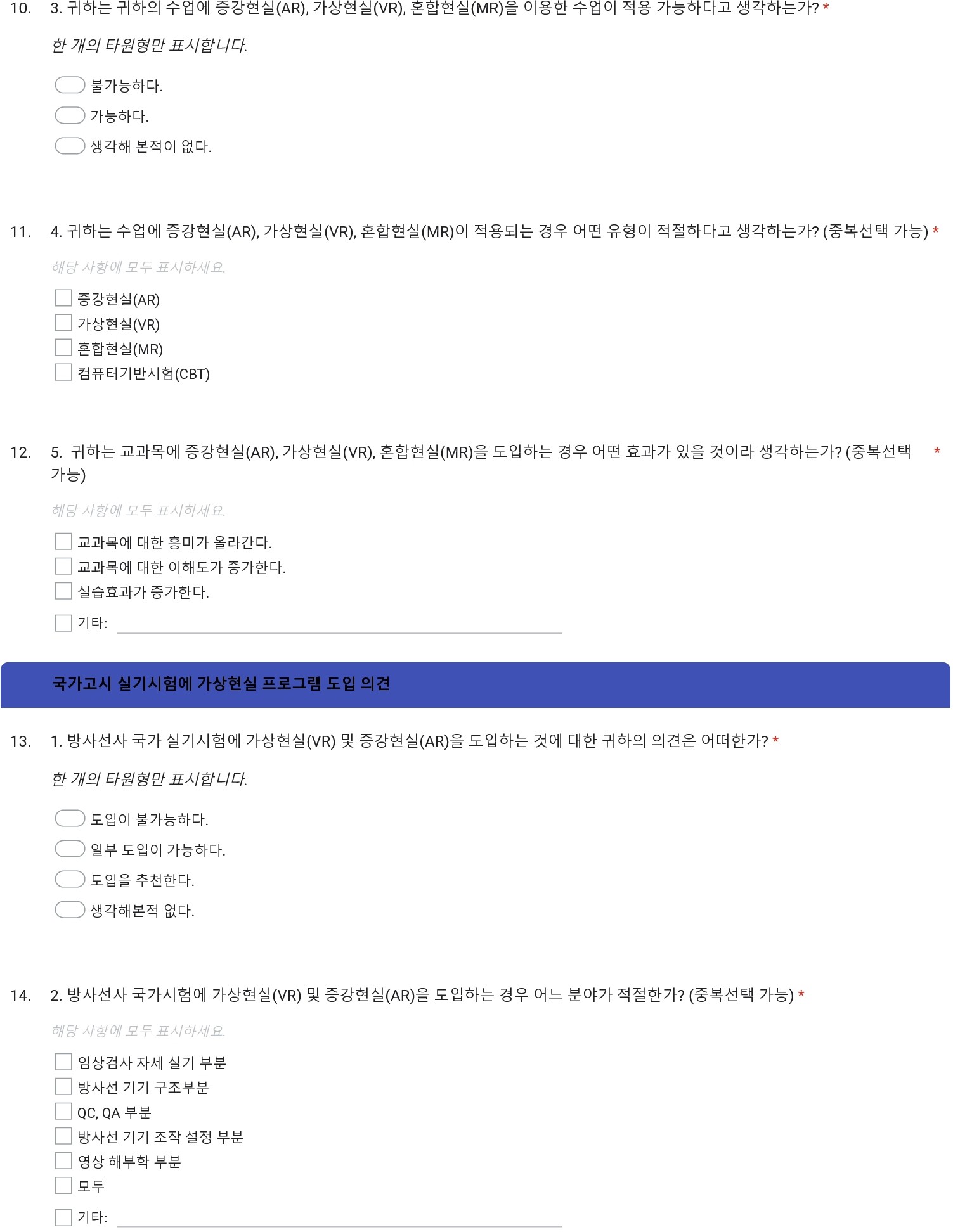


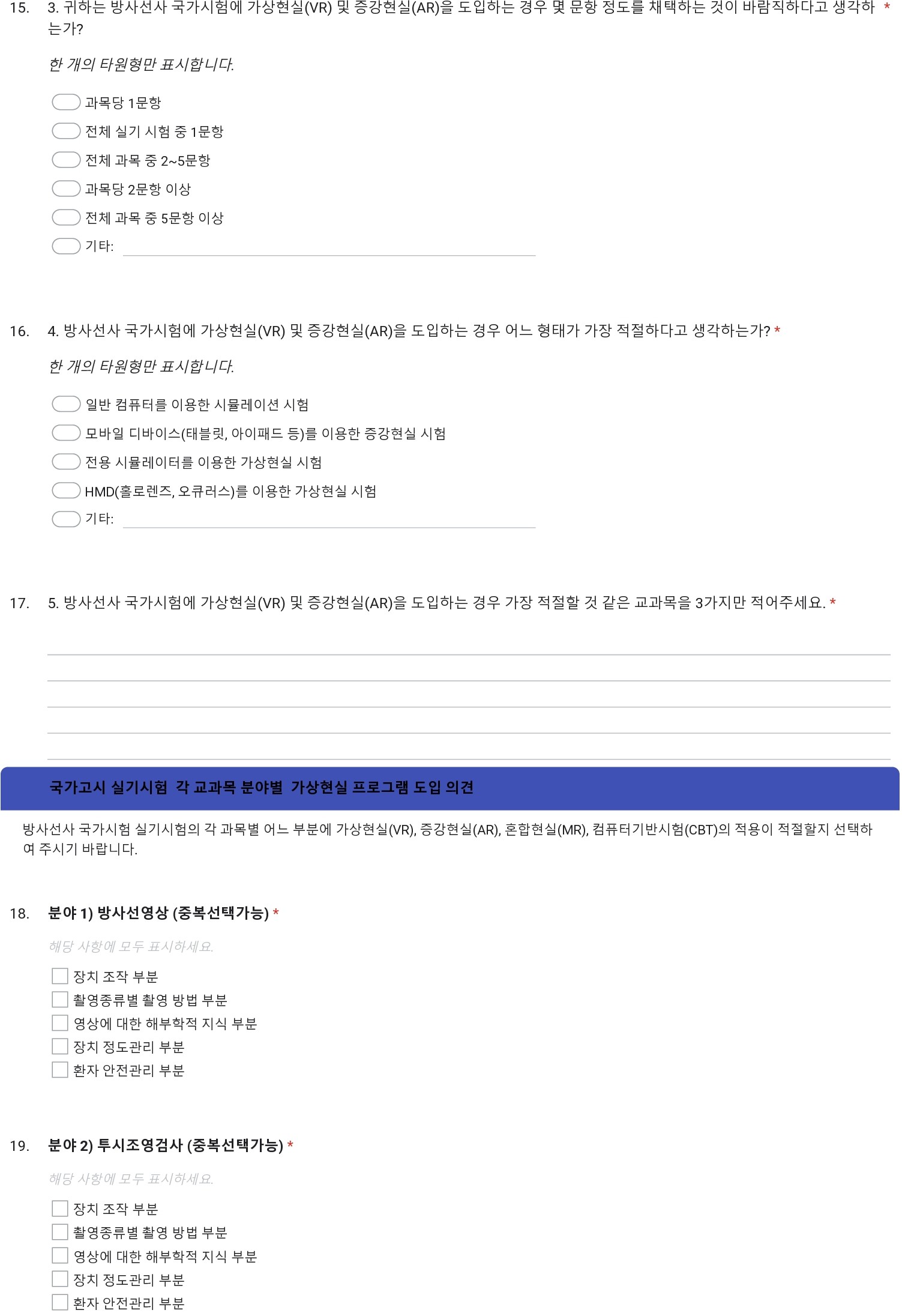


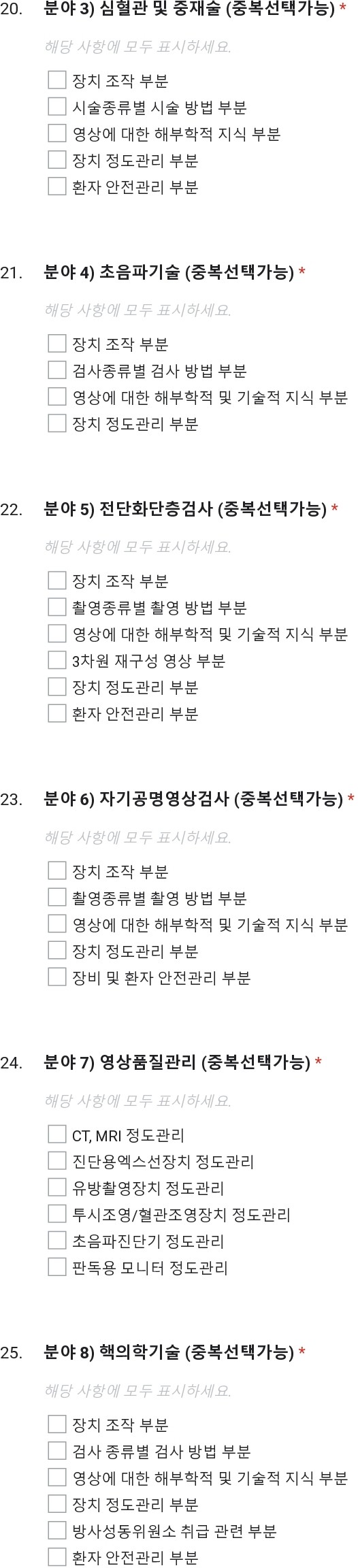


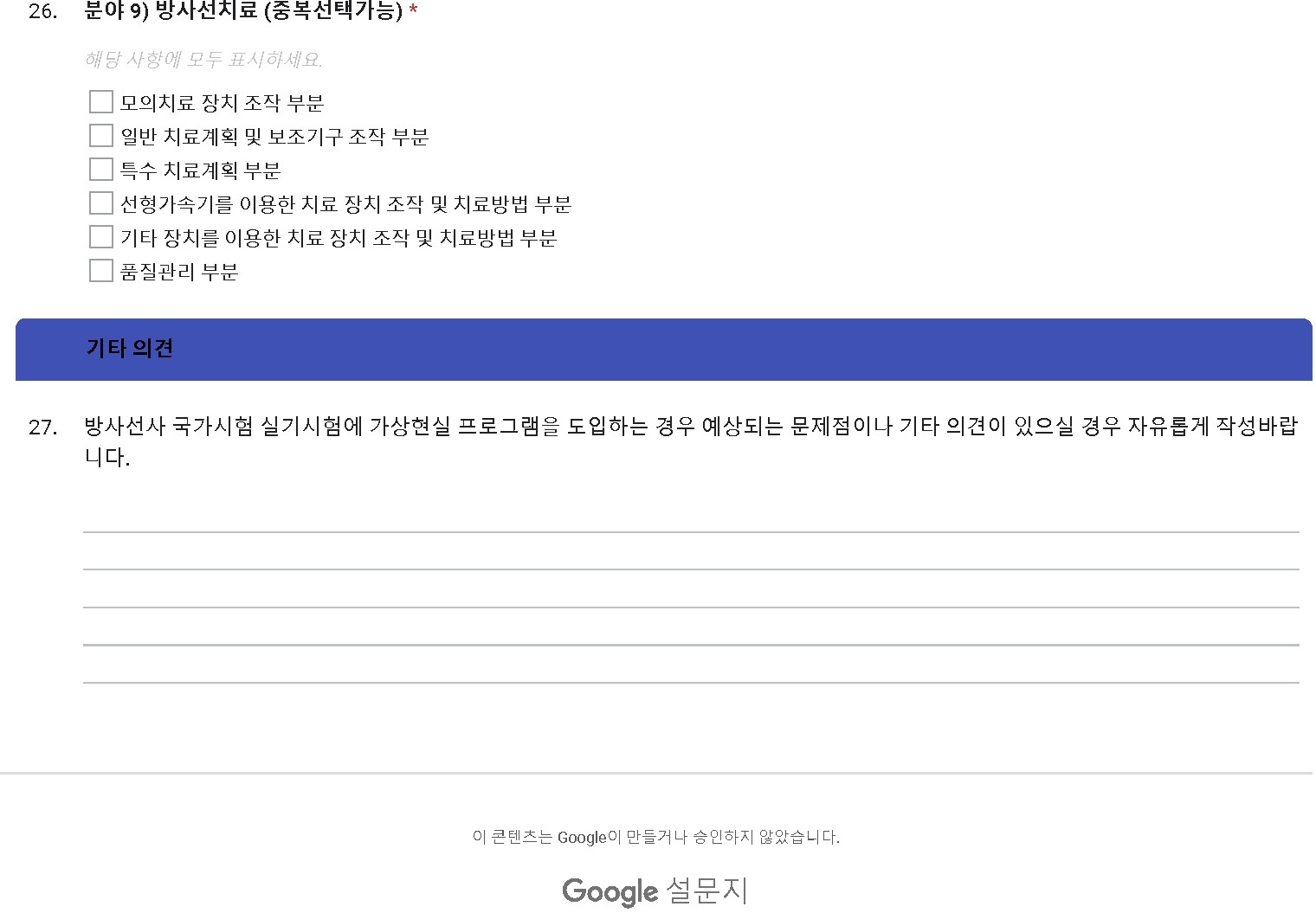

Supplement: Supplementary file 2 — Supplement 1. Survey questionnaires to measure the radiologic technologist students’ perceptions of the feasibility of incorporating VR programs into the practical tests of the national examination. [file jeehp-20-33-suppl1.docx]
